# Classification of Apricot Varieties by Infrared Spectroscopy and Machine Learning

**DOI:** 10.1021/acsagscitech.5c00068

**Published:** 2025-07-08

**Authors:** Jaume Béjar-Grimalt, David Pérez-Guaita, Ángel Sánchez-Illana, Rodolfo García-Contreras, Rashmi Kataria, Sylvie Bureau, Miguel de la Guardia, Frédéric Cadet

**Affiliations:** † Department of Analytical Chemistry, 16781 University of Valencia, 46100 Burjassot, Spain; ‡ Departamento de Microbiología y Parasitología, Facultad de Medicina, Universidad Nacional Autonoma de Mexico, 04510 Mexico City, Mexico; § School of Bioscience and Technology (SBST), Vellore Institute of Technology (VIT), 632 014 Vellore, Tamil Nadu, India; ∥ INRAE, Avignon University, UMR408 SQPOV, F-84000 Avignon, France; ⊥ Artificial Intelligence Department, PEACCEL, 75013 Paris, France

**Keywords:** Prunus armeniaca L, regression and classification, PLS-DA, SVM, RF, ATR–FTIR

## Abstract

This work aimed to investigate using ATR–FTIR spectroscopy combined with machine learning to classify eight apricot varieties. Traditionally, variety identification relies on physicochemical property measurements, which are time-consuming and require laboratory analysis. Instead, we used the ATR–FTIR spectra from 731 apricots divided into calibration (512) and test (219) sets and three machine learning models (i.e., partial least-squares-discriminant analysis (PLS-DA), support vector machine (SVM), and random forest (RF)) to accurately predict 97% of the test samples. Additionally, careful inspection of the PLS-DA regression vectors revealed a strong correlation between the spectra and biochemical composition in sugar and organic acids, validating ATR–FTIR spectroscopy as a viable alternative for variety identification. Finally, to validate the results, additional models were constructed using the physicochemical data from the apricots. These reference models were then tested using the same data splits as the spectroscopic data used as a reference method, obtaining similar results with both approaches.

## Graphical abstract

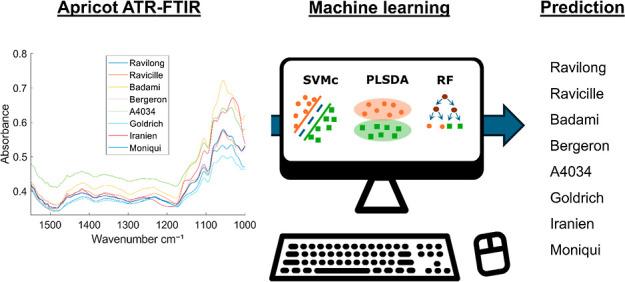

## 1 Introduction

According to the Food and Agriculture Organization (FAO), the apricot stands out as one of the most consumed temperate tree fruits, primarily cultivated in Mediterranean climates, with global production reaching 3.8 million tons in 2022.^1^ It is worth noting that the term “apricot” is rather generic and includes four different species and one naturally occurring interspecific hybrid: L., L, , and × ^2^. However, this classification is becoming increasingly complex due to the emergence of new cultivar varieties derived from crosses between varieties of different groups, developed to meet the consumer’s quality criteria.^3–5^ Simultaneously, there is a push to develop genotypes that can be cultivated in a broad area, since most apricot varieties are highly specific to their ecological requirements.^6^ Thus, the growth of new varieties requires the development of fast and reliable techniques to effectively characterize and identify different apricot fruit types.

Some of the methodologies used to identify apricot varieties are usually based on the determination of their physicochemical properties, such as sugar and carboxylic acid^7^ content, as well as their shape, length, and width.^8,9^ Molecular characterization methodologies based on DNA markers, such as simple sequence repeats^2^ or restriction fragment length polymorphism^10^ are also being used for the classification of different apricot cultivars. Although these approaches are useful for identifying the apricot variety, they are either time-consuming and/or require the use of reagents and intensive sample processing. Therefore, they generally need to be performed with equipment that can only be used in a laboratory by specialized personnel.

Fourier-transform infrared spectroscopy (FTIR) attenuated total reflectance (ATR) enables fast and cost-effective measurement with a minimal, if any, sample treatment, making it highly suitable for in situ analysis by regular users without specific expertise. In this context, ATR–FTIR has been extensively applied in food analysis,^11–13^ for the identification of different wheat varieties^14^ or the determination of the quality properties of virgin olive oils,^15^ as well as to obtain information about structural and chemical heterogeneity of fruits and vegetables,^16^ and has already been employed for the evaluation of apricot fruit biochemical composition,^17^ and their rheological parameters^18^ such as firmness, which is one of the principal parameters affecting apricot fruit quality. It is worth noting that ATR–FTIR spectroscopy can be used to analyze fresh and freeze-dried without significant differences as shown by Lan et al.,^19^ making it easier to implement in the fruit industry. Nevertheless, the spectra obtained from ATR–FTIR are highly complex due to the presence of a large number of overlapped bands that encode the biological information related to the fruit’s chemical composition. Fortunately, the coupling of ATR–FTIR with chemometrics and machine learning algorithms classifiers has proven capable of extracting biochemical markers from the IR variables. These techniques include typical linear methodologies such as partial least-squares-discriminant analysis (PLS-DA) as well as more sophisticated nonlinear methods such as support vector machine (SVM) and random forest (RF). The use of these methods facilitates the classification of different samples, as demonstrated in previous studies. For example, different fruits can be classified using various linear and nonlinear algorithms through image analysis,^20^ and adulterated foods can be identified using machine learning^21–23^ or authenticated in cases of protected designation of origin products.^24^

This study aimed to evaluate the capability of the ATR–FTIR to discriminate between different apricot varieties using supervised machine learning tools. To this end, the IR spectra data set obtained in a previous study^17^ was used to build and optimize calibration models that are capable of classifying the apricots by their variety. Results were then validated using an independent validation set and compared to those obtained using physicochemical properties to prove the utility of the ATR–FTIR spectra for the direct classification of different apricot varieties without requiring the use of reagents, in accordance with the green analytical chemistry criteria.^25^

## 2 Material and Methods

### 2.1 Samples and Data Acquisition

The spectral data set used in this work was produced by Bureau et al.^17^ Prior to the data analysis 20 samples were removed due to inconsistences in fruit maturity (e.g., some fruits were unusually green or small). All data, including spectra, chemical, and rheological properties, were acquired from a vast collection of 731 apricot fruits including eight varieties or hybrids, including 118 “Moniqui,” 102 “Goldrich,” 157 “Bergeron,” 66 “Iranien,” 17 “Badami,” 108 “Ravicille,” 24 “Ravilong,” and 140 “A4034,” at different maturity stages.

As described by Bureau et al.,^17^ nondestructive. A few days later, ATR–FTIR measurements and subsequently conventional destructive measurements were conducted. This resulted in a data set containing 23 measured properties and an FTIR spectrum for every fruit. A comprehensive overview of the observed physicochemical properties is described by Bureau et al.^17^

### 2.2 Chemometric Treatment and Methods

Preliminary analysis, PLS-DA, and SVM analysis were carried out using MATLAB R2023a from MathWorks (Natick, MA), employing the PLS_Toolbox functions from eigenvector (Manson, WA), and in-house written functions, scripts, and workflows. The data were split into two subsets for a test set using the onion algorithm from eigenvector^26^ into a calibration set consisting of 512 samples (70% of the samples), and a test set containing 219 samples (the remaining 30% of samples). The calibration data were utilized to build classification models employing PLS-DA with up to 10 latent variables (LVs). All models underwent cross-validation using the venetian blinds algorithm using 10 splits, and the cross validation (CV) classification error average was utilized to select the optimal number of latent variables based on the most probable prediction. The regression vectors were inspected to assign spectral features to potential chemical markers for correlation with the measured physicochemical properties.

Classification models based on SVM were also constructed using the calibration data. The radial basis function (RBF) was selected as the kernel function of SVM model training to better find nonlinear relationships in the data. For the γ SVM parameter, the grid ranges were 15 values, from 10^–6^ to 10, in spaced uniformly in log10. For the penalty parameter C, 10 values were used, also logarithmically spaced (base 10) from 10^–6^ to 102. These values correspond to by default by the PLS_Toolbox software for the hyperparameter optimization, which is designed to cover a broad and representative range of values on a logarithmic scale. This grid represents a practical compromise between sufficiently broad coverage and avoiding overly fine-tuning. A PLS compression was carried on based on the results from the previous PLS-DA to reduce variance and optimize the model.

Regarding preprocessing, typical normalization and derivative methods were evaluated. The final selection of preprocessing steps was based on two main criteria: low cross-validation error and interpretability of the regression vector. First, normal variate scaling normalization (SNV) was applied, which compensates for differences in absorbance arising from variations in the effective contact between the sample and the ATR crystal. Second, Savitzky–Golay smoothing and second order derivative computation (polynomial order: 2 window width:15) was employed to resolve convoluted bands with shoulders. Finally, the spectra were mean-centered to compensate for the different intensity of the bands and focus on the fluctuation of the data instead. After creating and optimizing the models with the calibration set, generalization capabilities and robustness of the models were evaluated using the test set. As reference labels for both, the IR and physicochemical data, we used the labels indicated by the different suppliers of the apricots. Classification errors, confusion matrix and receiver operating characteristic curve (ROC) obtained in the independent testing were employed to evaluate the model’s performance.

Additionally, Orange Data Mining Toolbox^27^ version 3.36.2 developed by the Bioinformatics Lab (Ljubljana, Slovenia), was used to perform Random Forest (RF) classified following the same preprocessing as in PLS-DA and SVM. These models were selected since they are widely employed models that fall into the category of lineal (PLS-DA) and nonlineal (SVM and RF) models, to compare the performance of each group, since the FTIR spectroscopy is susceptible to various interfering factors such as scattering, reflection, and interference, which manifest themselves as nonlinear artifacts such as baseline or band distortion.^28^ All the raw data employed in this work together with a MATLAB live script and an Orange workflow containing the data analysis are available as a data set in the Zenodo repository.^29^

## 3 Results and Discussion

### 3.1 Classification of Apricot Varieties Using PLS-DA

The employed initial classification method was PLS-DA. Considering CV results, 8 LVs were selected, as increasing the number of LVs provided only a small improvement in the classification error and could introduce unwanted variance to the model. The selected LVs explained 99% of the variance of the data, and the obtained model had an average cross validation classification error of 2.7%. The independent validation set was subsequently classified using the generated model. Figure 1 depicts the calculated *Y* values for each class together with the binary classification threshold. This threshold is calculated using a Bayesian approach according to the PLS_Toolbox implementation^30,31^ which is based on the probability of belonging to a specific class. As it is shown, the model successfully discriminated most of the varieties. Furthermore, samples were assigned to the class where they achieved the highest probability score (i.e., “most probable approach”) to construct the corresponding confusion matrix (see Table 1). As a result, out of the 213 samples used for independent testing, only 7 were misclassified, including 5 Goldrich samples classified as Ravilong, 1 as Bergeron samples, and 1 Bergeron classified as Goldrich. In total, more than 95% of the samples were correctly predicted. The AUC observed in the ROC curves (Figure S1) for each of the classes was 0.99 for the Ravilong, Ravicille, Badami, Bergeron, A4034, and Moniqui varieties, 0.96 for the Goldrich variety, 1 for the Iranien one, corroborating the ability to greatly distinguish between varieties.

**Figure 1:**
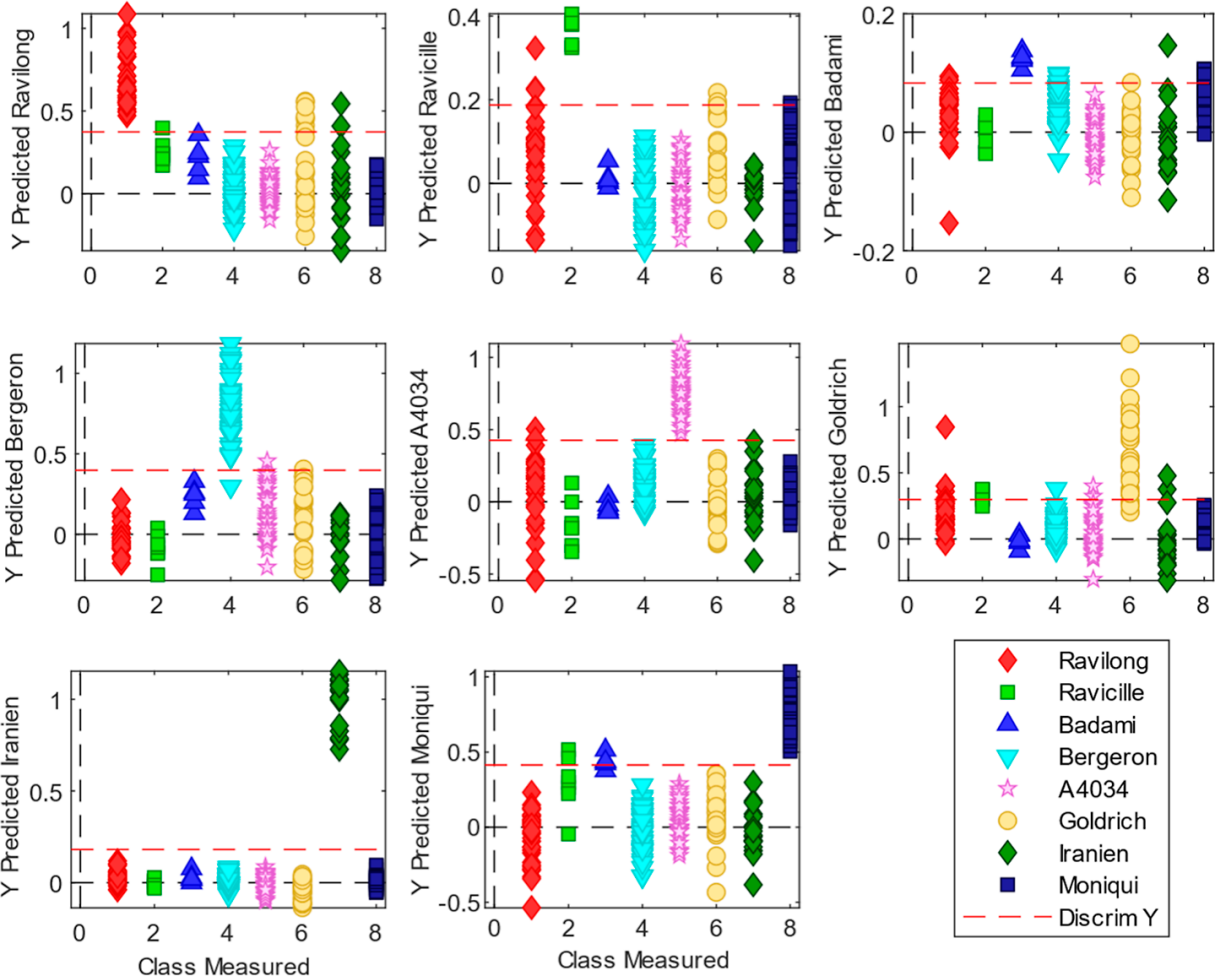
PLS-DA classification results using the model built with the apricot spectra. The graphs show calculated *Y* values for the PLS-DAs performed for each class versus the measured being for the different samples (test set). The red dashed line stands for the calculated binary threshold by the PLS_Toolbox implementation of PLS-DA.^30^

**Table 1:** Confusion Matrix for the PLS-DA Classification Model Used to Predict the Apricot Variety from the ATR–FTIR Data (Test Set)

	actual class							
	Ravilong	Ravicille	Badami	Bergeron	A4034	Goldrich	Iranien	Moniqui
predicted as Ravilong	**35**	0	0	0	0	**5**	0	0
predicted as Ravicille	0	**7**	0	0	0	0	0	0
predicted as Badami	0	0	**5**	0	0	0	0	0
predicted as Bergeron	0	0	0	**50**	0	**1**	0	0
predicted as A4034	0	0	0	0	**41**	0	0	0
predicted as Goldrich	0	0	0	**1**	0	**22**	0	0
predicted as Iranien	0	0	0	0	0	0	**18**	0
predicted as Moniqui	0	0	0	0	0	0	0	**35**

### 3.2 Classification of Apricot Varieties Using Nonlinear Approaches

Furthermore, the same data set was evaluated using nonlinear machine learning techniques to assess whether they improved the errors obtained by the PLS-DA. First, classification models were built using SVM classification. The SVM hyperparameters C and γ, selected using CV error as a target value, were set to 100 and 0.0032, respectively. To eliminate unwanted sources of variation in the data and reduce the dimensionality, data was preprocessed with a PLS compression algorithm using 8 LVs following the same reasoning as for the PLS-DA. The classification error on the calibration set was 0.5%, while the CV classification error was 1.59%, indicating that the model performs consistently across training and validation and is unlikely to be overfitted. The model was then tested using the same independent test set as in the PLS-DA. Figure 2 clearly shows that SVM achieved a more effective separation for each apricot variety compared with the previous PLS-DA model, and the global model, which was also based on the “most probable” approach, being able to assign 100% of the samples to their real classes as seen in the confusion matrix (Table 2). The AUC observed in the ROC curves (Figure S2) for each of the classes was 0.99 for the Badami variety and 1 for the rest, showing again better results than the ones obtained employing PLS-DA.

**Figure 2:**
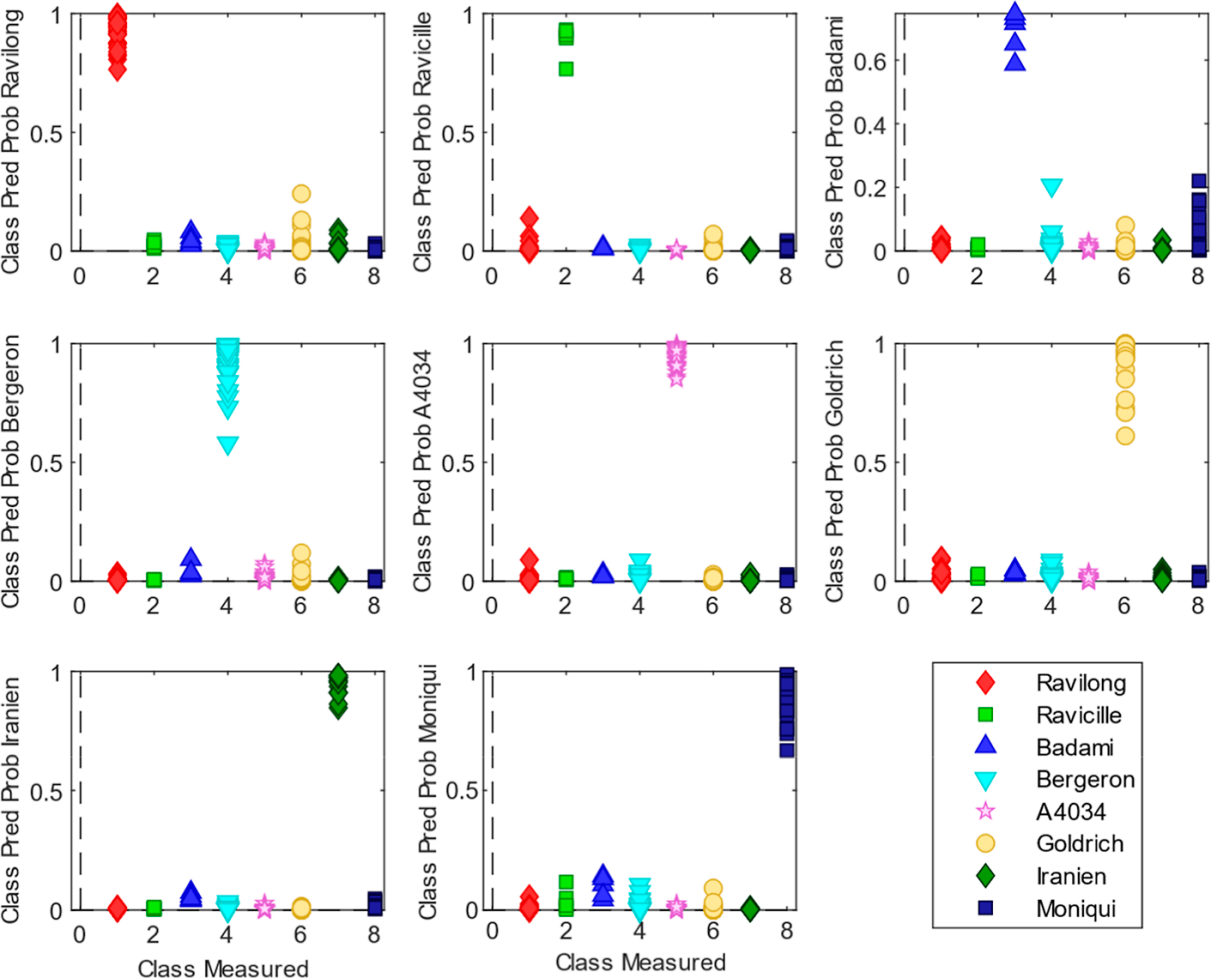
SVM classification results using the model built from apricot spectra showing the class prediction probability versus the class measured (test set).

**Table 2:** Confusion Matrix for the SVM Model Used to Predict the Apricot Variety from ATR–FTIR Spectra (Test Set)

	actual class							
	Ravilong	Ravicille	Badami	Bergeron	A4034	Goldrich	Iranien	Moniqui
predicted as Ravilong	**35**	0	0	0	0	0	0	0
predicted as Ravicille	0	**7**	0	0	0	0	0	0
predicted as Badami	0	0	**5**	0	0	0	0	0
predicted as Bergeron	0	0	0	**51**	0	0	0	0
predicted as A4034	0	0	0	0	**41**	0	0	0
predicted as Goldrich	0	0	0	0	0	**27**	0	0
predicted as Iranien	0	0	0	0	0	0	**18**	0
predicted as Moniqui	0	0	0	0	0	0	0	**35**

Second, RF was also used to classify the samples. A total of 128 trees were used to build the models, as increasing the number of trees beyond this point did not yield significant performance gains. This choice was based on the findings of T.M. Oshiro et al.,^32^ who reported that median AUC values show minimal improvement beyond 64 trees and no significant differences after 128 trees. Therefore, 128 trees represent a good balance between AUC performance, processing time, and memory usage. For the Random Forest model, the calibration error was 0%, and the CV classification error was 0.3%. Additionally, RF also obtained a classification accuracy of 100% as can be seen in the confusion matrix obtained (Table 3), and an AUC of 0.99 for the Ravicille variety, and 1 for all the other varieties (Figure S3), evidencing that the prediction models based on nonlinear approaches such as SVM and RF demonstrated superior results compared to the PLS-DA model in classifying the different apricot varieties. In general, FTIR variables follow a linear relationship with the concentration of the components of the fruits (i.e., Beer–Lambert law). However, in spectra acquired through ATR, some experimental issues can distort this linearity, such as changes in the effective path length due, for example, to ineffective contact with the ATR crystal. Most importantly, it should be noted that the changes in sample composition associated with each variety are not necessarily linear, which can explain the improvement obtained by nonlinear models.

**Table 3:** Confusion Matrix for the Random Forest Classification Model Used to Predict the Apricot Variety from the ATR–FTIR Data (Test Set)

	actual class							
	Ravilong	Ravicille	Badami	Bergeron	A4034	Goldrich	Iranien	Moniqui
predicted as Ravilong	**35**	0	0	0	0	0	0	0
predicted as Ravicille	0	**7**	0	0	0	0	0	0
predicted as Badami	0	0	**5**	0	0	0	0	0
predicted as Bergeron	0	0	0	**51**	0	0	0	0
predicted as A4034	0	0	0	0	**41**	0	0	0
predicted as Goldrich	0	0	0	0	0	**27**	0	0
predicted as Iranien	0	0	0	0	0	0	**18**	0
predicted as Moniqui	0	0	0	0	0	0	0	**35**

While results clearly indicate that the use of such approaches is preferable for prediction purposes, SVM and RF have limitations in terms of the interpretability of the model. For example, it is hard to establish which FTIR variables, and by extension, which molecular components of fruit, are involved in the classification. In principle, this information does not improve model performance but proves valuable for understanding which variables—in this case, spectral bands—differ for each cultivar, how they contribute to the model, and how they relate to their physicochemical properties. This can be useful, for example, when evaluating the potential effects of interferences. in the model. Unfortunately, this task is harder to accomplish with RF and SVM models due to their “black box” nature. Variable selection methods have been proposed to study the importance of the different variables.^33–35^ In general, these methodologies, such as kernel principal component analysis (KPCA), rank the different variables according to relevance and eliminate the ones with less importance. Although these sophisticated algorithms are designed to provide better classification results with less complex models, they can also be used to study the importance of each wavenumber.

A simpler approach consists of the use of linear models. For example, PLS-DA holds an advantage over SVM and RF in providing easily accessible information about the importance of variables used for classification. Several model parameters, such as the variable importance in projection (VIP) scores^36^ or the selectivity ratio can provide straightforward knowledge about the importance of variables. In addition, the regression vector also provides information about the direction of each variable in the classification. In the next section, we studied the relationship between the different wavenumbers and the classification using the regression vector of the PLS-DA.

### 3.3 Regression Vectors Study

The mean spectra for each apricot variety colored with the values of the regression vectors for the PLS-DA model can be seen in Figure 3, illustrating the differences in contribution from the spectra to the model for each variety, in which differences in the spectra for each apricot variety are already obvious. The spectral range used for the analysis covered the region between 1000 and 1550 cm^–1^, which corresponds to the absorption region of the apricot’s major components, sugars, and organic acids.^17^ The set of overlapped major bands within the 1053–904 cm^–1^ region is assigned to the C–O and C–C stretching modes present in carbohydrates,^37^ while the bands located within the 1474–1199 cm^–1^ region are assigned to the O–C–H,

**Figure 3:**
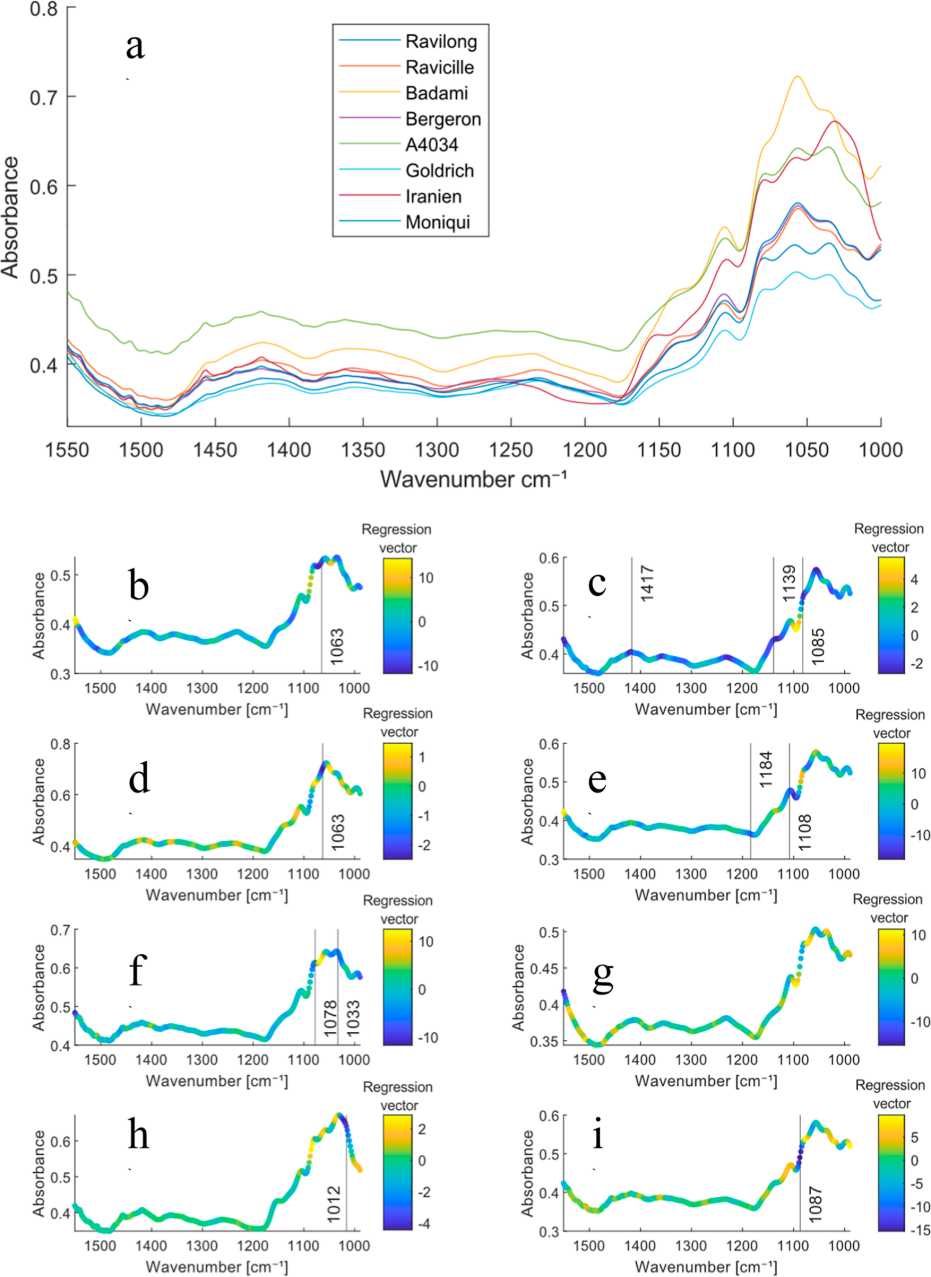
(a) Mean spectra of each apricot variety, and mean spectra colored with the value of the regression vector (warm colors indicate positive values and cold colors indicate negative values) for each variety being (b) Ravilong, (c) Ravicille, (d) Badami, (e) Bergeron, (f) A4034, (g) Goldrich, (h) Iranien, (i) Moniqui.

C–C–H, C–O–H stretching modes.^38^ It must be noted that, since the PLS-DA was performed using the second derivative, maximum negative contributions (i.e., blue colors) in the regression vector relate to intense absorbance in the spectra. For the apricot variety Ravilong (Figure 3b), a substantial contribution at 1063 cm^–1^ can be seen, which can be related to the presence of fructose, being its key band.^38^ The Ravicille (Figure 3c), variety presents a strong value for the regression vector at 1085 cm^–1^, which can be assigned to the δ_a_COH vibration of the citric acid.^39^ It has strong contributions all over the region going from 1417 to 1139 cm^–1^ where the citric acid presents various robust absorbance bands, which agrees with the measured mean chemical values for every variety the Table 4, which shows that Ravicille is the one with the highest content in citric acid. The Badami (Figure 3d) variety, in the same way as with the Ravilong, presents a high value for its regression vector at 1063 cm^–1^, which as stated beforehand, is a key peak of the infrared spectrum of the fructose.^40^ This also correlates with the chemical parameters depicted in the Table 4 since the Badami is the variety with the highest content of fructose. The Bergeron variety (Figure 3e) presents a positive contribution from the regression vector at 1108 cm^–1^, which can be related to the ν­(C–OH) vibration mode which is strong in the malic acid alongside 1184 cm^–1^,^41^ which can also be appreciated in the regression vector, being consistent with the Bergeron variety being the one with the highest malic acid concentration. The A4034 variety (Figure 3f) presents high values at 1078 cm^–1^, and 1033 cm^–1^, which correspond to the major peak of the glucose,^38^ which is the major sugar for this variety. The Goldrich variety (Figure 3g), on the other hand, does not present hight values in the C–O region when compared to the other varieties, being the variety with the lower content in sugar. Lastly, the Iranien variety (Figure 3h) presents a strong contribution to the regression vector at 1012 cm^–1^, which correlates with the highest absorption band of the sucrose,^42^ the main sugar present in the variety, with the highest value among all of them, and the Moniqui (Figure 3i) presents a strong contribution at 1087 cm^–1^. From this, we can deduce that the varieties with higher content in sugars and organic acids rely on the primary bands for those compounds for their classification, while the other classes rely on the combination of other spectral features. It is worth noting that this demonstrates the ability of PLS-DA to link the results obtained to the original data, allowing for further exploration compared to nonlinear methods. Due to the multivariate nature of these methods, even though the highest contribution comes from certain bands, the model still utilizes all of them to some extent, hence increasing the method robustness.

**Table 4:** Mean Values for the Biochemical Composition in Sugars and Organic Acids of Every Apricot Variety, Obtained from the Values Registered by Bureau et al.^17^

sample	glucose (g 100 g^–1^ FW)	fructose (g 100 g^–1^ FW)	sucrose (g 100 g^–1^ FW)	citric acid (meq 100 g^–1^ FW)	malic acid (meq 100 g^–1^ FW)
Iranien	2.57 ± 0.32	1.16 ± 0.18	8.04 ± 1.53	0.62 ± 0.39	0.09 ± 0.49
Badami	2.44 ± 0.25	1.47 ± 0.11	8.01 ± 1.79	22.19 ± 2.60	10.02 ± 1.04
Moniqui	1.84 ± 0.27	0.80 ± 0.12	5.68 ± 2.30	19.38 ± 5.53	6.59 ± 0.63
Bergeron	2.05 ± 0.25	0.70 ± 0.13	5.33 ± 1.12	8.13 ± 1.17	21.19 ± 3.71
A4034	2.86 ± 0.78	0.80 ± 0.25	4.39 ± 1.40	9.86 ± 3.01	12.37 ± 3.84
Ravicille	1.42 ± 0.28	0.47 ± 0.08	5.54 ± 0.77	28.86 ± 2.53	4.98 ± 0.59
Ravilong	2.17 ± 0.59	1.12 ± 0.30	2.72 ± 0.84	26.61 ± 6.20	5.24 ± 0.69
Goldrich	1.40 ± 0.34	0.56 ± 0.17	3.70 ± 1.02	22.23 ± 5.87	9.79 ± 2.52

On the other hand, looking at the regression vector values for each apricot variety, we can see that there is a notable difference between the misclassified samples. As stated previously, 1 Bergeron was classified as Goldrich, and 5 Goldrich as Ravilong with 1 being classified as Bergeron. When looking at the spectra colored with the regression vector, we can appreciate that they present a very different regression vector, with different contributions all around the spectra. This could indicate that the reason some samples were misclassified is because they were in a different ripening stage (samples were selected depending on the dates of harvesting), having variations in their content in sugars that led to the misclassification.

### 3.4 Comparison of the Models Developed with that Obtained from Physicochemical Parameters Classification

The results obtained from the PLS-DA, SVM, and RF classification methods built using the ATR–FTIR spectra were subsequently validated. This was achieved by generating PLS-DA, SVM, and RF classification models utilizing the physicochemical properties, following a similar approach as before. The outcomes obtained from these models were then compared with those obtained using FTIR spectroscopy. The models were constructed using the same calibration and validation data sets as before. Similarly to the ATR–FTIR based models, 8 LVs were selected for the PLS-DA, resulting in a CV classification error of 1.3% and an AUC of 0.99 for the Ravilong, Ravicille, A4034, Goldrich, and Moniqui, and 1 for the Badami, Bergeron, and Iranien (Figure S4). This error is slightly lower than that obtained with the ATR–FTIR based PLS-DA. The results for the classification of the validation set are depicted in Figure S5 of the Supporting Information. The confusion matrix (Table S1) evidenced that only 3 samples were misclassified, two samples belonging to the Ravilong group were predicted as Goldrich, and one sample belonging to Ravicille was predicted as Ravilong, resulting in a classification error of 1.41%. The SVM model built from the sample physicochemical parameters (see Figure S6 and Table S2), accurately classified all the samples except 1 Ravilong, which was misclassified as a Ravicille, with an AUC of 1 for all the varieties. This performance was slightly inferior to the model obtained using the FTIR spectra. Lastly, the RF model obtained using the physicochemical parameters achieved perfect classification just as the one obtained using the FTIR spectroscopy (Table S3) with an AUC of 1 for every variety. The comparison between the confusion matrix of validation sample variety obtained through PLS-DA, SVM, and RF evidence the good comparability between models built from ATR–FTIR spectra and those obtained from the complete characterization of the physicochemical parameters of samples, thus offering a reagent free, fast, and cost-effective procedure for the screening of apricot variety.

As evidenced by these results, ATR–FTIR spectroscopy coupled with supervised machine learning algorithms provides a useful methodology for the identification of apricot varieties. Three approaches, PLS-DA, SVM, and RF, were used for the modeling of the spectral data, and the different methodologies were validated using an independent test set consisting of 213 samples. The validation of the PLS-DA showed a classification error of 3.3%, while the models based on nonlinear methods, SVM and RF, improved that figure to 0% and did not feature any misclassification. Moreover, while PLS-DA has an edge in the interpretability of the information about the samples used for classification and its variables as loadings, it is worth noting that we only discussed well-known groups already covered in the literature, avoiding unfounded assumptions. However, the spectral data is composed of many more points spanning the entire spectral region, which also contribute to the model. The purpose was to demonstrate that, despite PLS-DA showing worse results than nonlinear models, it has the advantage of being able to correlate with the initial data. In general, these results prove that the combined use of chemometrics and ATR–FTIR was able to find differences among the different varieties and use those differences to successfully identify each sample with its corresponding class. On the other hand, it should be noted that the exceptionally accurate classification results were obtained using a validation set with a limited number of samples (213). While encouraging, applying this technique to broader practical contexts will require extensive validation with larger data sets. Future efforts will focus on increasing considerably the sample size test, enabling the evaluation of the impact of temporal and instrument-to-instrument variability on the results. In conclusion, it can be said that FTIR spectroscopy provides not only an excellent means for the visualization of the chemical composition of the apricots, but is also a fast, reliable, and cost-effective way for the estimation of their different varieties.

## Supplementary Material



## Notes

ROC curves for PLS-DA, SVM, and Random Forest models, classification results for apricots based on physicochemical properties, and confusion matrix for the physicochemical models (PDF)
