# Supplementary material for: Classification of Apricot Varieties by Infrared Spectroscopy and Machine Learning
Source: ACS Agric Sci Technol. 2025 Jul 8;5(7):1373–81. doi: 10.1021/acsagscitech.5c00068 (PMC12309246; doi:10.1021/acsagscitech.5c00068)
Supplement: Supplementary file 1 [file as5c00068_si_001.pdf]

Supporting Information to

Classification of apricot varieties by infrared spectroscopy and machine learning

Jaume Béjar-Grimalt[1], David Pérez-Guaita[1*], Ángel Sánchez-Illana[1*], Rodolfo Garcia Contreras[2], Rashmi Kataria[3], Sylvie Bureau[4], Miguel de la Guardia[1], Frédéric Cadet[5]

[1]Department of Analytical Chemistry, University of Valencia, Burjassot, Spain.
[2]Departamento de Microbiología y Parasitología, Facultad de Medicina, Universidad Nacional Autonoma de Mexico, Mexico City, Mexico.

[3]School of Bioscience and Technology (SBST), Vellore Institute of Technology (VIT), Vellore, Tamil Nadu, India.

[4]INRAE, Avignon University, UMR408 SQPOV, F-84000 Avignon, France
[5]PEACCEL, Artificial Intelligence Department, Paris, France.

*Corresponding Author: david.perez-guaita@uv.es and angel.illana@uv.es

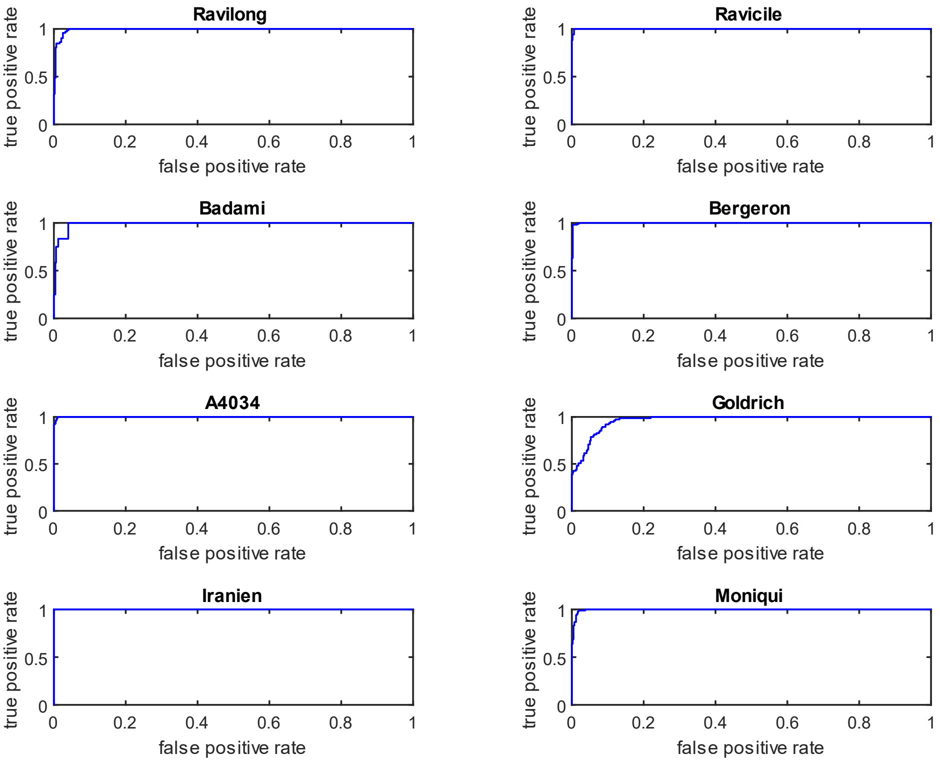

Figure S1. ROC curves for the PLS DA classification models calculated employing the spectral data in MATLAB

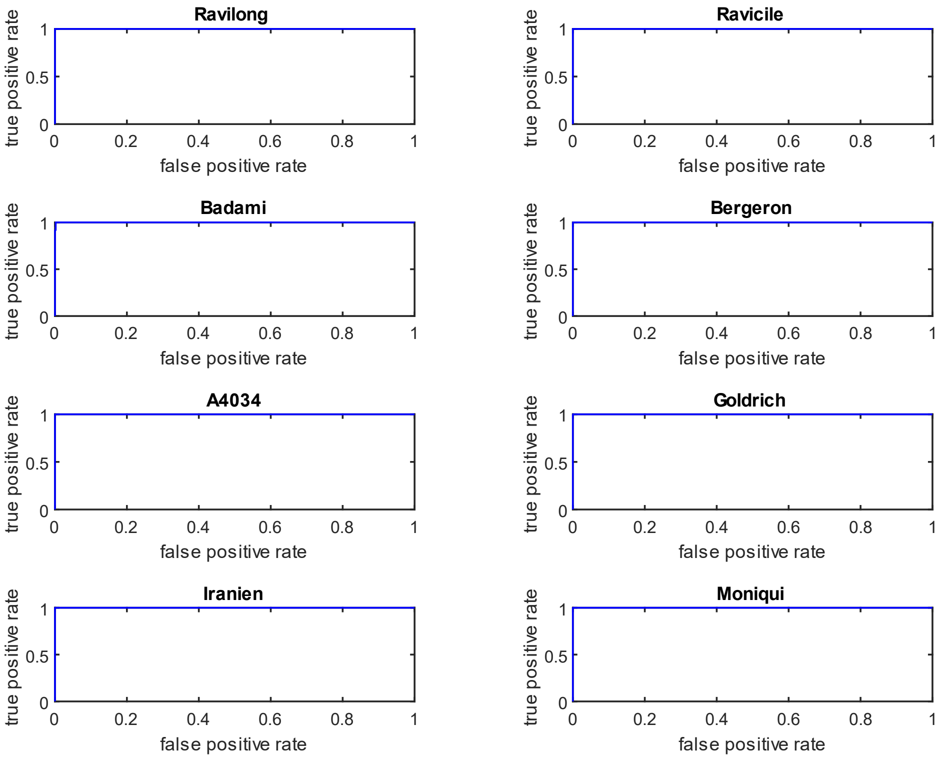

Figure S2. ROC curves for the SVM classification models calculated employing the spectral data in MATLAB

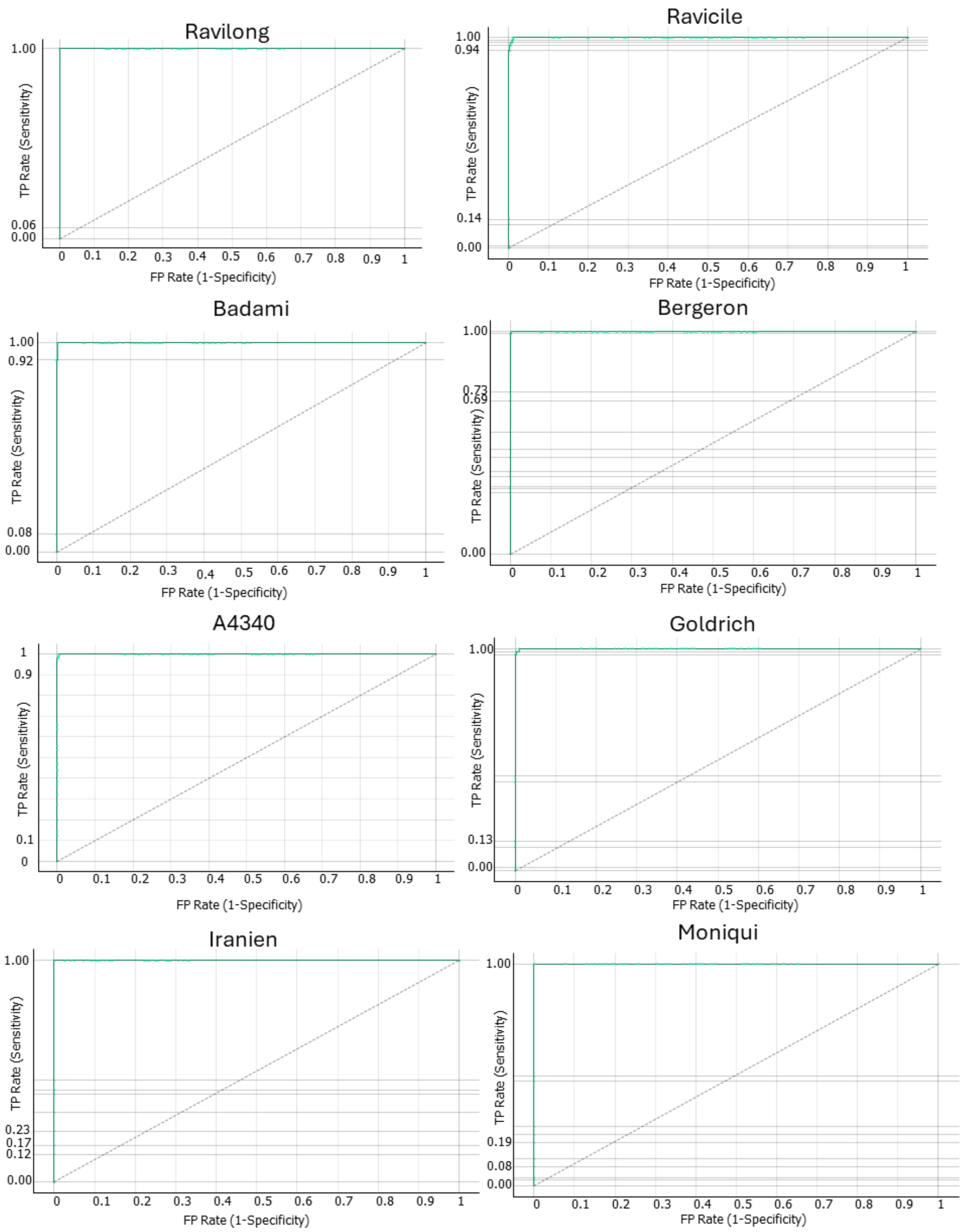

Figure S3. ROC curves for the random forest classification models calculated employing the spectral data in orange datamining.

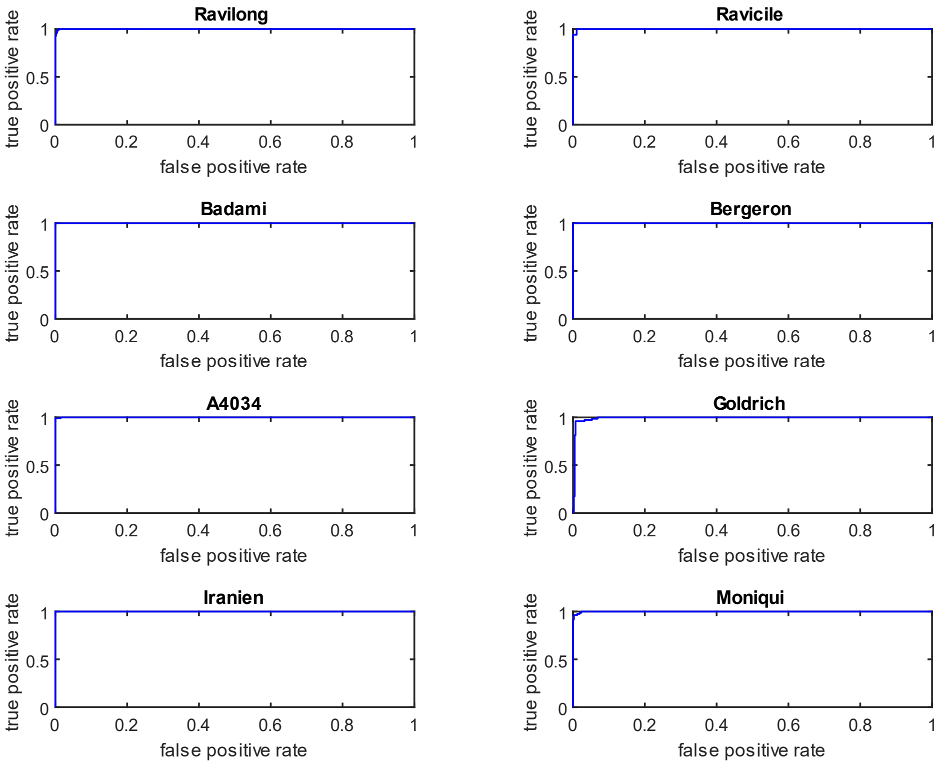

Figure S4. ROC curves for the PLS DA classification models calculated employing the physicochemical data in MATLAB

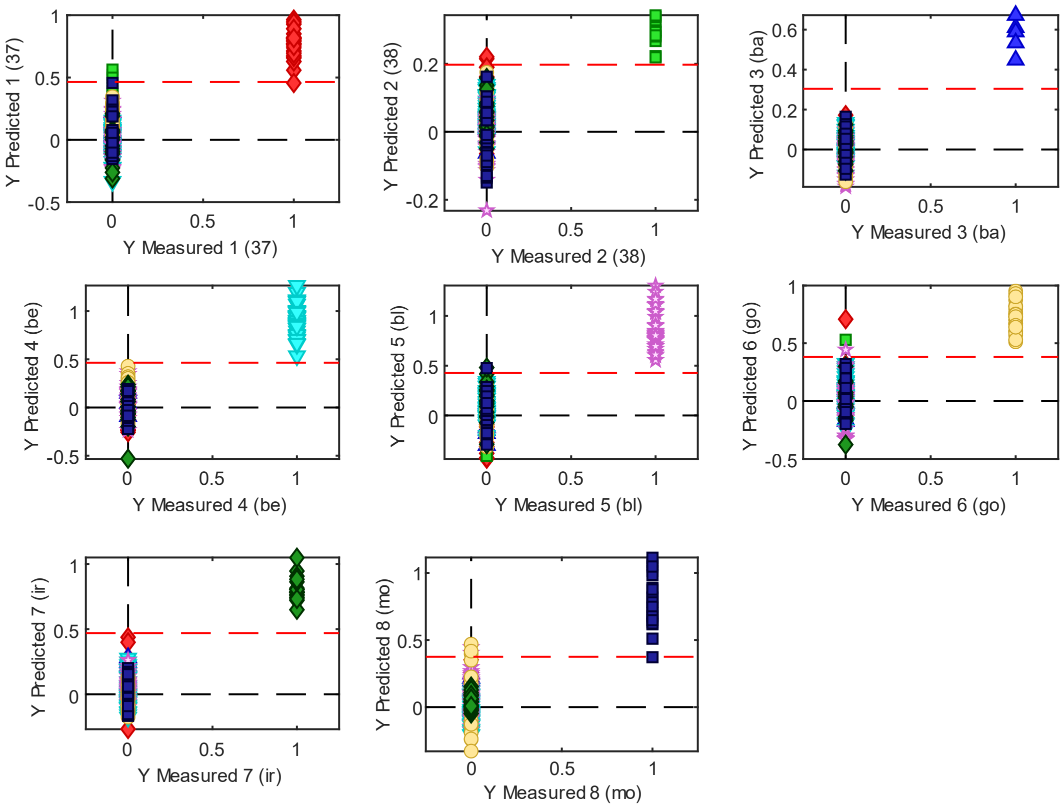

Figure S5. Results for the classification of the apricot variety using a PLS-DA model built from the physicochemical parameters of samples.

Table S1. Confusion matrix for the PLS-DA model used to predict the apricot variety from the physicochemical data.

	**Actual class**							
	Ravilong	Ravicille	Badami	Bergeron	A4034	Goldrich	Iranien	Moniqui
Predicted as Ravilong	**35**	0	0	0	0	0	0	0
Predicted as Ravicille	0	**9**	0	0	0	0	0	0
Predicted as Badami	0	0	**5**	0	0	0	0	0
Predicted as Bergeron	0	0	0	**50**	0	0	0	0
Predicted as A4034	0	0	0	0	**40**	0	0	**1**
Predicted as Goldrich	**1**	**1**	0	0	0	**33**	0	0
Predicted as Iranien	0	0	0	0	0	0	**19**	0
Predicted as Moniqui	0	0	0	0	0	0	0	**25**

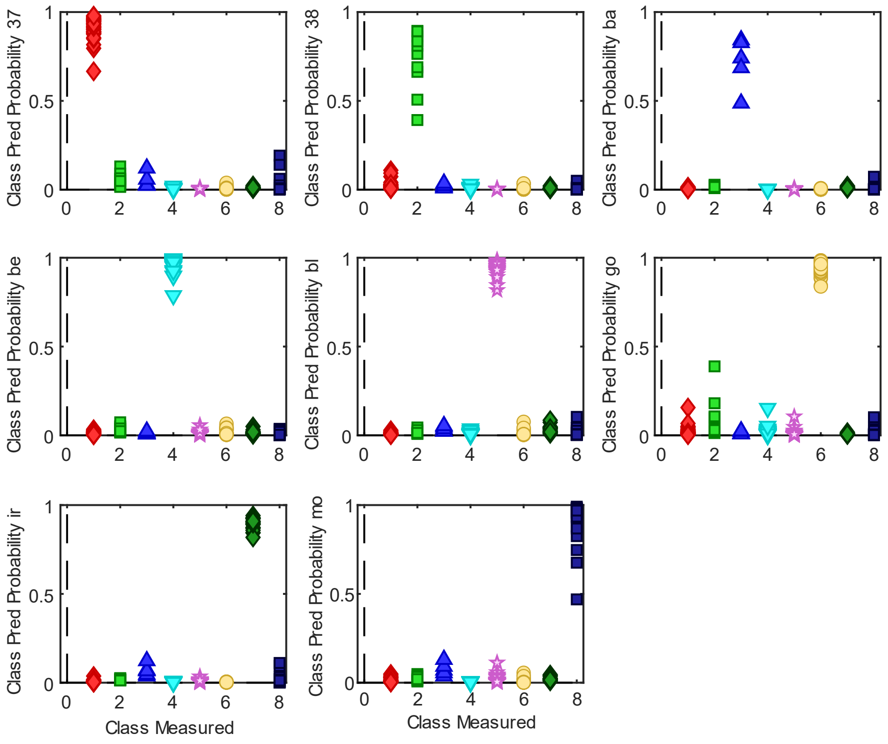

Figure S6. Results for the classification of the apricot variety using the SVMc built with the physicochemical parameters.

Table S2. Confusion matrix for the SVMc model used to predict the apricot variety from the physicochemical data.

	**Actual class**							
	Ravilong	Ravicille	Badami	Bergeron	A4034	Goldrich	Iranien	Moniqui
Predicted as Ravilong	**35**	0	0	0	0	0	0	0
Predicted as Ravicille	**1**	**10**	0	0	0	0	0	0
Predicted as Badami	0	0	**5**	0	0	0	0	0
Predicted as Bergeron	0	0	0	**50**	0	0	0	0
Predicted as A4034	0	0	0	0	**40**	0	0	0
Predicted as Goldrich	0	0	0	0	0	**33**	0	0
Predicted as Iranien	0	0	0	0	0	0	**19**	0
Predicted as Moniqui	0	0	0	0	0	0	0	**26**

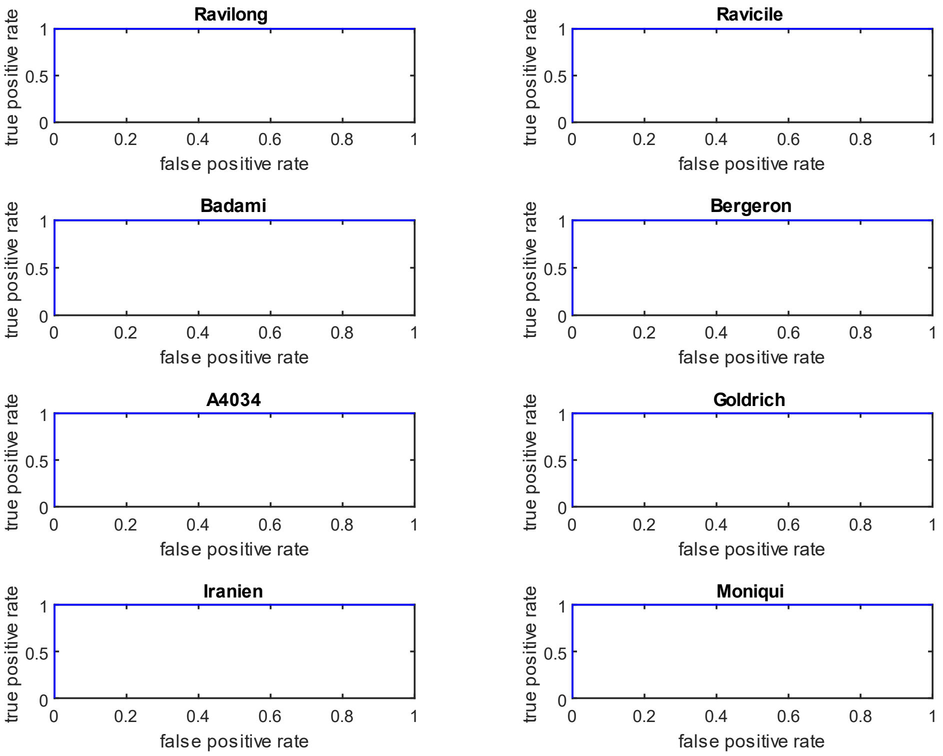

Figure S7. ROC curves for the SVM classification models calculated employing the physicochemical data in MATLAB

Table S3. Confusion matrix for the random forest model used to predict the apricot variety from the physicochemical data.

	**Actual class**							
	Ravilong	Ravicille	Badami	Bergeron	A4034	Goldrich	Iranien	Moniqui
Predicted as Ravilong	**35**	0	0	0	0	0	0	0
Predicted as Ravicille	0	**7**	0	0	0	0	0	0
Predicted as Badami	0	0	**5**	0	0	0	0	0
Predicted as Bergeron	0	0	0	**51**	0	0	0	0
Predicted as A4034	0	0	0	0	**41**	0	0	0
Predicted as Goldrich	0	0	0	0	0	**27**	0	0
Predicted as Iranien	0	0	0	0	0	0	**18**	0
Predicted as Moniqui	0	0	0	0	0	0	0	**35**

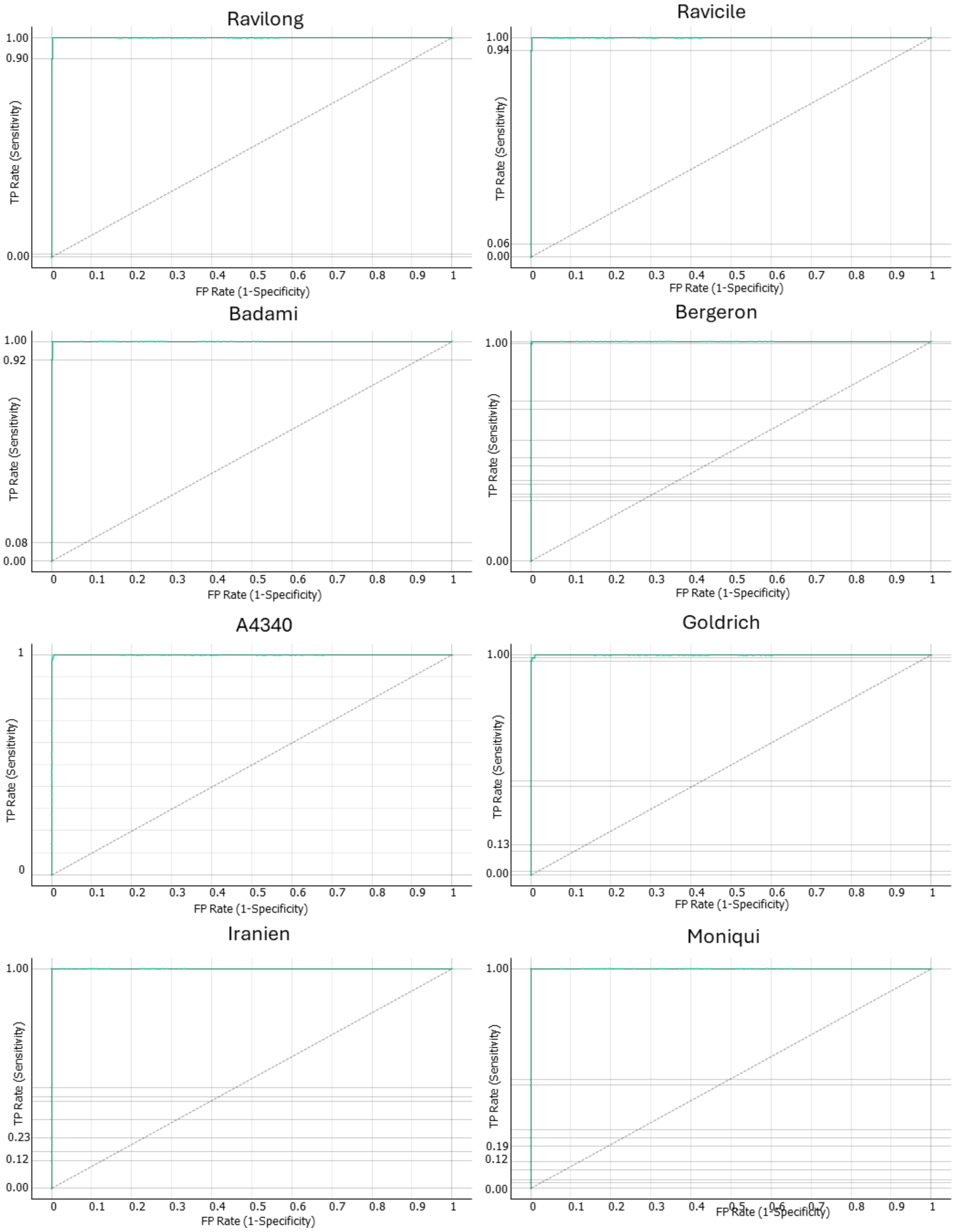

Figure S8. ROC curves for the random forest classification models calculated employing the physicochemical data in orange datamining.
